# Diagnostic Performance of Different Thyroid Imaging Reporting and Data Systems (Kwak-TIRADS, EU-TIRADS and ACR TI-RADS) for Risk Stratification of Small Thyroid Nodules (≤10 mm)

**DOI:** 10.3390/jcm9010236

**Published:** 2020-01-16

**Authors:** Simone Schenke, Rigobert Klett, Philipp Seifert, Michael C. Kreissl, Rainer Görges, Michael Zimny

**Affiliations:** 1 Division of Nuclear Medicine, Department of Radiology and Nuclear Medicine, Magdeburg University Hospital, Leipziger Straße 44, 39120 Magdeburg, Germany; michael.kreissl@med.ovgu.de; 2 Institute for Nuclear Medicine Hanau/Giessen/Offenbach/Frankfurt, Paul-Zipp-Straße 171, 35398 Gießen, Germany; rigobert.klett@t-online.de (R.K.); zimny@nuk-hu.de (M.Z.); 3 Clinic of Nuclear Medicine, Jena University Hospital, Am Klinikum 1 A4U1, 07740 Jena Lobeda-Ost, Germany; Philipp.seifert@med.uni-jena.de; 4 Department of Nuclear Medicine, Essen University Hospital, Hufelandstraße 55, 45122 Essen, Germany; rainer.goerges@uni-due.de

**Keywords:** TIRADS, small thyroid nodules, thyroid carcinomas, risk stratification

## Abstract

Due to the widespread use of ultrasound, small thyroid nodules (TNs) ≤ 10 mm are common findings. Standardized approaches for the risk stratification of TNs with Thyroid Imaging Reporting and Data Systems (TIRADS) were evaluated for the clinical routine. With TIRADS, the risk of malignancy in TNs is calculated by scoring the number or combination of suspicious ultrasound features, leading to recommendations for further diagnostic steps. However, there are only scarce data on the performance of TIRADS for small TNs. The aim was to compare three different TIRADS for risk stratification of small TNs in routine clinical practice. We conducted a retrospective cohort analysis of TNs ≤ 10 mm and their available histology. Nodules were classified according to three different TIRADS. In the study, 140 patients (*n* = 113 female) with 145 thyroid nodules (*n* = 76 malignant) were included. Most of the malignant nodules were papillary carcinoma (97%), and the remaining 3% were medullary carcinoma. For all tested TIRADS, the prevalence of malignancy rose with increasing category levels. The highest negative predictive value was found for ACR TI-RADS and the highest positive predictive value for Kwak-TIRADS. All tested variants of TIRADS showed comparable diagnostic performance for the risk stratification of small TNs. TIRADS seems to be a promising tool to reliably assess the risk of malignancy of small TNs.

## 1. Introduction

Due to the widespread use of ultrasound as well as the increasing number of imaging studies performed for reasons other than planned assessment of the thyroid gland (i.e., computed tomography of the chest or cervical spine, magnetic resonance imaging of the cervical spine, and whole-body positron emission tomography), incidental thyroid nodules (TNs) ≤ 10 mm are now common findings [1,2,3,4,5]. The characterization of TNs has improved because of better ultrasound image resolution and the introduction of new diagnostic ultrasound tools such as elastography [6]. Furthermore, standardized approaches for the risk assessment of TNs, so-called Thyroid Imaging Reporting and Data Systems (TIRADS), have been evaluated for routine clinical use. With TIRADS, the risk of a malignant TN is calculated by scoring the number or the combination of various suspicious ultrasound criteria [7,8,9]. Recently, a number of standardized reporting systems (e.g., ACR TI-RADS, EU-TIRADS, Korean-TIRADS) have been proposed. These reporting systems also include recommendations for the appropriate further diagnostic steps [7,8,9]. For instance, the ACR TI-RADS recommends a fine-needle-aspiration biopsy (FNA) for highly suspicious TNs if the diameter is 10 mm or larger. Otherwise, the ACR is in concordance with other guidelines, which do not generally recommend FNA for the evaluation of nodules smaller than 10 mm [9,10]. The EU-TIRADS, published in 2017, recommends shared decision-making (FNA vs. active surveillance) with the patient when subcentimetric TNs with highly suspicious ultrasound features without abnormal lymph nodes are detected [8]. However, there are only scarce data on the performance of TIRADS for small TNs up to a diameter of 10 mm. The purpose of this study was to compare three different variants of TIRADS (Kwak-, ACR, and EU-TIRADS) in terms of the risk stratification of small TNs (≤10 mm) in the routine clinical practice.

## 2. Experimental Section

This monocentric cohort analysis was approved by the local ethical committee (Magdeburg University Hospital, No. 43/19) and the need for a written informed consent was waived. Data collection with standardized thyroid ultrasound started in 2015 for all consecutive patients referred to our outpatient practice for the assessment of TNs found at neck ultrasound or other imaging modalities, or suspected thyroid dysfunction. Inclusion criteria for this cohort analysis were the presence of thyroid nodules ≤ 10 mm measured in B-mode ultrasound (independent of the histopathological size of the nodule), Kwak-TIRADS classification of the thyroid nodule during ultrasound investigation between 2015 and 2017, and available histopathological results after surgery. Only the categorization of the TNs according to ACR TI-RADS and EU-TIRADS was performed retrospectively using the (electronically) archived ultrasound images. We did not include incidental thyroid cancers detected at final histology and TNs > 10 mm measured with ultrasound. The malignant TNs were histopathologically classified according to the 8th UICC edition of TNM classification and modified according to Schmid et al. 2018, where pT1a2 carcinomas replaces pT3 ≤ 10 mm with minimal extrathyroidal extension [11,12]. According to the German guidelines, thyroid scans (data not shown) were performed only if one or more nodules had a diameter of ≥ 10 mm or in patients with a low TSH level [13]. Due to the retrospective design of this cohort study, there were various reasons for thyroid surgery, such as to exclude the malignancy of TNs with suspicious findings on ultrasound, FNA, or ^99m^Tc-MIBI imaging; thyroid nodules accompanied by suspicious cervical lymph nodes or growth during follow-up; the patient’s wish for a definite histopathological diagnosis; or local symptoms related to the thyroid.

Thyroid ultrasound was performed by three qualified investigators with two to five years of experience in Kwak-TIRADS classification and more than 7 years each in thyroid ultrasound using B-mode ultrasound with a linear probe with a frequency of 8–13 MHz (HITACHI Avius Hi Vision, Chiyoda, Japan). For all nodules, the composition (completely solid, almost completely solid, 10–50% cystic changes, >50% cystic proportion), echogenicity (hypoechogenicity, marked hypoechogenicity, isoechogenicity, hyperechogenicity), margins (well circumscribed, irregular, spiculated), presence of internal calcification (hyperechoic spots, microcalcifications, macrocalcifications, none), and the orientation of the nodule (taller-than-wide, wider-than-tall) was documented. Additionally, the size of each nodule was assessed.

Kwak-TIRADS calculates a score as the sum of ultrasound characteristics that are present in the TNs of interest. The number of suspicious ultrasound features (e.g., solid or almost solid nodule, hypoechogenicity, irregular margins, presence of microcalcifications, and a taller than wide shape, respectively) are used to reveal a score of TIRADS 3, 4A, 4B, 4C, or 5. As the number of suspicious features increases, so does the risk of malignancy [7]:

TIRADS 3: no suspicious features (risk 1.7%);
TIRADS 4A: one suspicious feature (risk 3.3%);
TIRADS 4B: two suspicious features (risk 9.2%);
TIRADS 4C: three or four suspicious features (risk 44.4–72.4%);
TIRADS 5: five suspicious features (risk 87.5%).

EU-TIRADS defines four ultrasound features of high suspicion for malignancy (non-oval or round shape, irregular margins, microcalcifications, and a marked hypoechogenicity) [8]:

EU-TIRADS 2: anechoic or entirely spongiform (benign, risk 0%);
EU-TIRADS 3: entirely isoechoic or hyperechoic (low risk, risk 2–4%);
EU-TIRADS 4: mildly hypoechoic (intermediate risk, risk 6–17%);
EU-TIRADS 5: at least one of the four features of high suspicion (high risk, risk 26–87%).

ACR TI-RADS is a more sophisticated scoring system with five ultrasound features (composition, echogenicity, shape, margin, and echogenic foci). Each feature is described in detail and weighted by allocating points to create a summed score [9]:

TR1: 0 points, benign (aggregate risk level 0.3%);
TR2: 2 points, not suspicious (aggregate risk level 1.5%);
TR3: 3 points, mildly suspicious (aggregate risk level 4.8%);
TR4: 4–6 points, moderately suspicious (aggregate risk level 5.9–12.8%);
TR5: 7 points or more, highly suspicious (aggregate risk level 20.8–68.4% for 10 points).

In our study we defined the cutoffs as suspicious for malignancy for Kwak-TIRADS ≥ 4C, EU-TIRADS > 4, and ACR TI-RADS ≥ 4, respectively.

### 2.1. Pathological Examination

The tissue samples were fixed in 4% formaldehyde solution. After dehydration they were embedded in paraffin. Slices with a thickness of 5 µm were stained using hematoxylin-eosin. In the case of follicular neoplasia, additional cuts were performed and EvG (Elastica van Gieson stain) was applied. Microscopic assessment of the slices was performed in 20-fold to 400-fold enlargements.

### 2.2. Statistics

All statistical tests were performed using WinSTAT for Microsoft^®^ Excel version 2005.1. The results were expressed as mean, standard deviation (SD), median, and 25th/75th percentile, respectively. The variables were tested using the *t*-test and the Mann–Whitney test as indicated. Adjusting for multiple comparisons using Bonferroni correction, results were considered to be significant if a *p*-value of <0.01 was found.

## 3. Results

We included 140 patients (*n* = 113 female (81%) with a mean age of 46.3 ± 11.4 years and *n* = 27 male with a mean age of 58.2 ± 12.6 years) with 145 TNs (*n* = 76 malignant nodules (52%), mean size 7.6 ± 1.9 mm and *n* = 69 benign nodules, mean size 7.8 ± 1.8 mm, *p* = 0.608). Most of the malignant nodules were papillary thyroid carcinomas (PTC, 97%), and the remaining 3% were medullary carcinomas (MTC). Of the carcinomas, 55 (72%) were pT1a1 tumors and 9 (12%) were pT1a2 tumors. Twelve malignant nodules (16%) were classified as pT1b tumors in final histopathology because the histopathological size was larger than the size measured by sonography (*n* = 12/76, 15.7%). Lymph node metastases (LNMs) were found in 20% of the PTC and there were no LNMs in the two cases of MTC (Table 1). Histopathology revealed multifocality in 39% of the PTCs. Malignant nodules were significantly more often solitary nodules than benign nodules (*p* < 0.001).

For all examined variants of TIRADS, with increasing TIRADS category the prevalence of malignancy also increased. The prevalence of malignancy for Kwak-TIRADS classes 3, 4A, 4B, 4C, and 5 was 0%, 0%, 16.7%, 67.9%, and 81%, respectively. In EU-TIRADS for classes 2–5 the malignancy rate was 0%, 4.2%, 8.3%, and 67.9%, whereas the prevalence of malignancy for ACR TI-RADS classes TR1–TR5 was 0%, 0%, 0%, 56.1%, and 69.7%, respectively (Table 2).

**Figure 1:**
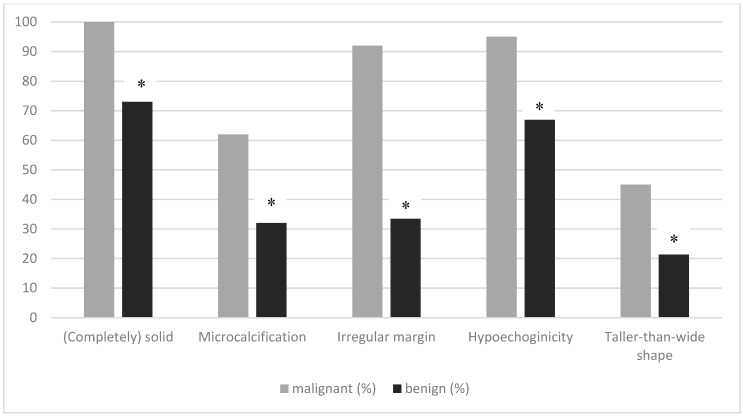
Percentage of various suspicious ultrasound features for the differentiation of malignant from benign thyroid nodules. Univariate analysis showed significant differences between malignant and benign TNs for all suspicious features (* *p* < 0.01).

All of the malignant nodules were solid or almost completely solid (benign TNs: 73%). The malignant nodules were hypoechoic (95%), the margins were more often irregular (92%), and they showed more microcalcifications (62%) compared to the benign ones (67% hypoechoic, 36% irregular margins, and 32% microcalcifications). All suspicious features documented at thyroid ultrasound were significantly more frequent in malignant nodules than in benign nodules (Figure 1). The diagnostic performance values of TIRADS are listed in Table 3. The highest negative predictive value to rule out malignancy was found for ACR TI-RADS. The positive predictive value was comparable for all tested variants of TIRADS; all systems showed low specificity (Table 3).

## 4. Discussion

The management of small TNs is widely debated because the clinical relevance of papillary thyroid microcarcinoma remains controversial [10,14,15,16,17]. Since the introduction of ultrasound reporting systems for TNs in 2009 by Horvath et al., the development of different TIRADS increased continuously [18]. Common to all these TIRADS is the standardized acquisition of ultrasound features, which raise the suspicion of malignancy to allow a risk stratification of the TNs. Furthermore, the risk class is linked with a recommendation for further diagnostic workup depending on the nodules’ sizes [7,8,9,10]. Recently, many studies have been published validating certain TIRADS for TNs > 10 mm as described in a meta-analysis by Castellana et al. [19]. However, data are rare regarding the diagnostic performance of TIRADS concerning TNs with a maximum size of 10 mm. This study compares the three most commonly used TIRADS in small TNs. Our study demonstrates that 97% of the malignant TNs presented with a high-risk Kwak-TIRADS (4C or 5) or EU-TIRADS (EU 5) classification and that 100% presented with an ACR TI-RADS TR4 or 5. These high sensitivities are comparable to the results of Du et al., where the Kwak-TIRADS alone and in combination with ultrasound elastography was tested in small TNs [20]. However, contrary to our study they summarized Kwak-TIRADS 4B–5 as malignant, because the prevalence of malignancy for Kwak-TIRADS 4B was remarkably high: 70.5% vs. 16.7% in our study and 9.2% in the data from the original paper of Kwak et al. The accuracy for Kwak-TIRADS alone was found to be 83.8%, slightly higher than our results for Kwak-TIRADS. This major discrepancy in TIRADS 4B accuracy might be caused by a center-specific selection bias. Considering the distribution of the ultrasound features, Du et al. described that most of the malignant TNs were solid, hypoechoic, and showed irregular margins, comparable to our results. However, we found a lower percentage of malignant TNs with a taller-than-wide shape than Du et al. (45% versus 58%) and a higher percentage of microcalcifications in malignant TNs (61% versus 44%). Another study by Mendes et al. examined whether Kwak-TIRADS is useful for the assessment of subcentimetric TNs. The nodules were divided into two groups (2–5 mm and 6–10 mm, respectively). In accordance with our results, they demonstrated that the rate of malignancy increased with the number of suspicious features, independent of the nodule size group [21]. Our results are also supported by a recent study by Ha et al. [22]. The authors evaluated the performance of different TIRADS (Korean-TIRADS, French-TIRADS, ATA scoring system, and a web-based system among others) in TNs smaller than 10 mm. The authors focused on the low malignancy rates of “low-suspicion” TIRADS classes. These results indicate that the use of TIRADS could prevent the overdiagnosis and overtreatment of “low-risk” TNs [22]. In our study, the rate of malignancy in the “low-risk” TIRADS classes (Kwak-TIRADS 3 and 4A, ACR TI-RADS TR1 and TR2, and EU-TIRADS EU 2 and 3, respectively) was 0% for Kwak-TIRADS and ACR TI-RADS, and 1.3% in EU-TIRADS. However, the proportion of nodules classified as “low-risk” was very small (Kwak-TIRADS 19%, ACR TI-RADS 8%, and EU-TIRADS 17%). This finding is likely caused by a selection bias in our study, which leads to a small number of cases with low TIRADS classes that were referred to thyroid surgery or FNA. In another study, Weiss et al. focused on the use of ACR TI-RADS for 61 subcentimetric TNs. As the gold standard, the results of FNA (Bethesda system) were used. Of five papillary carcinomas, all were classified as TR4/TR5 (“high-risk” nodules). Comparable to our results, of the nodules that presented with a low TIRADS score (TR1/TR2), 88% were benign on FNA (12% were nondiagnostic/unsatisfactory, none of them were malignant in Bethesda classification). They concluded that TIRADS may also be used for risk stratification of small TNs and that lesions with a low TIRADS score may be followed without the need for immediate FNA [23]. Interestingly, we underestimated the size of 16% of the PTC with B-mode sonography. These results are in concordance with other studies. Zhao et al. found rates of underestimation for TNs ≤ 10 mm of 8.3%, whereas Deveci et al. described rates of 14.3% [24,25]. In the study of Hahn et al. the tumor size agreement between sonographical and histopathological measurements was defined as a difference of less than 20% and they found a rate of underestimation of 13.2%. They stated that ultrasound size measurement is influenced by cystic changes and irregular margins of the nodules [26].

### Limitations

This study has several limitations. First, this study is a retrospective analysis and a selection bias is unavoidable. We started using TIRADS in daily routine in our outpatient practice and referral center in 2015. With increasing experience and continued use there was a shift of indication for surgery from “multinodular goiter with nodules” to “suspicious thyroid nodule”. A shared decision-making process together with the patients was introduced, especially for younger patients who asked for a definitive exclusion of malignancy by histopathology. Moreover, the small number of nodules in the “low-risk” classes as well as the very high percentage of malignant TNs in our cohort can be explained by this selection bias and the fact that nodules that appeared benign in the ultrasonographic examination were not referred to further diagnostic workup by FNA or surgery. Additionally, this selection bias may affect the negative and positive predicting value. Therefore, prospective studies with a more balanced distribution are desirable. Secondly, we also did not evaluate the interobserver variability of the different reporting systems in this study. In order to minimize the variability in the assessment of the sonographic features, image data were routinely reviewed by all participating physicians and TIRADS consensus building was established. Furthermore, we conducted a multicenter study to compare the interobserver variability of different imaging reporting systems (data not shown) [27]. Thirdly, the classifications according to ACR TI-RADS and EU-TIRADS were performed only by one experienced physician by reviewing the archived ultrasound images that were partly recorded by a different examiner. Thus, it cannot be excluded that not all relevant sonographic criteria were stored, particularly at the beginning of the standardized ultrasonography in 2015. Fourthly, most of the malignant nodules were papillary thyroid carcinomas. Therefore, the diagnostic performance in other types of thyroid cancer requires further investigation.

## 5. Conclusions

In our study, ACR TI-RADS with a cutoff ≥ TR4 showed the highest sensitivity and negative predictive value, whereas a Kwak-TIRADS score ≥ 4C had the highest positive predictive value and accuracy. TIRADS seems to be a promising clinical tool to reliably assess the risk of malignancy of small thyroid nodules, even in primary care settings, and to select thyroid nodules for further diagnostic workup. Furthermore, it might be considered to extend the follow-up time for small thyroid nodules and low-risk TIRADS classification.

## Figures and Tables

**Table 1:** Thyroid carcinomas (TC) and lymph node metastases stages.

	All TC *n* = 76	TC pT1a1 *n* = 55	TC pT1a2 *n* = 9	TC pT1b *n* = 12
pN0 n (%)	37 (48.7)	26 (47.2)	4 (44.5)	7 (58.3)
pN1 n (%)	15 (19.7)	9 (16.4)	3 (33.3)	3 (25.0)
pNX n (%)	24 (31.6)	20 (36.4)	2 (22.2)	2 (16.7)

**Table 2:** Distribution of malignant and benign thyroid nodules for all tested variants of Thyroid Imaging Reporting and Data Systems (TIRADS) classifications.

	Malignant Thyroid Nodules (*n* = 76, %)	Benign Thyroid Nodules (*n* = 69, %)	Prevalence of Malignancy (%)
Kwak-TIRADS			
3 n (%)	0 (0)	9 (13.1)	0
4A n (%)	0 (0)	19 (27.5)	0
4B n (%)	2 (2.6)	10 (14.5)	16.7
4C n (%)	57 (75.0)	27 (39.1)	67.9
5 n (%)	17 (22.3)	4 (5.8)	81.0
ACR TI-RADS			
TR1 n (%)	0 (0)	4 (5.8)	0
TR2 n (%)	0 (0)	8 (11.6)	0
TR3 n (%)	0 (0)	16 (23.2)	0
TR4 n (%)	23 (30.3)	18 (26.1)	56.1
TR5 n (%)	53 (69.7)	23 (33.3)	69.7
EU-TIRADS	0 (0)		
2 n (%)	0(0)	0 (0)	0
3 n (%)	1 (1.3)	23 (33.3)	4.2
4 n (%)	1 (1.3)	11 (15.9)	8.3
5 n (%)	74 (97.4)	35 (50.7)	67.9

**Table 3:** Diagnostic performance of Kwak-TIRADS, EU-TIRADS, and ACR TI-RADS.

	Sensitivity (%)	Specificity (%)	Positive Predictive Value (%)	Negative Predictive Value (%)	Accuracy (%)
Kwak-TIRADS 4C and 5	97.4	55.1	70.5	95	77.2
ACR-TI-RADS TR4 and TR5	100	40.6	65	100	71.7
EU-TIRADS 5	97.4	49.3	67.9	94.4	74.5
